# Supplementary material for: Impact of Seriphium plumosum densification on Mesic Highveld Grassland biodiversity in South Africa
Source: R Soc Open Sci. 2020 Apr 15;7(4):192025. doi: 10.1098/rsos.192025 (PMC7211835; doi:10.1098/rsos.192025)
Supplement: Permit MP [file rsos192025supp1.pdf]

Mpumalanga
TOURISM AND PARKS AGENCY

MPB. 5568

MPUMALANGA TOURISM & PARKS AGENCY
P/BAG X 11338, NELSPRUIT 1200 TEL: 013 750 5300/1
2017 -01- 12
FAX: 013 759 5490
WILDLIFE PROTECTION SERVICES
PERMITS

PERMIT

TO ~~HUNT~~/CATCH / COLLECT AND CONVEY ANIMALS FOR SCIENTIFIC PURPOSE

(Issued in terms of the provisions of the Nature Conservation Act 10 of 1998)

Name and residential address of permit holder: *S.C. Patrocinio*
Portion 49 JR Farm Sterkfontein,
Suite 332, Private Bag x 7294
Witbank 1035

Name and address of institution or department of whose behalf shall be hunted and conveyed: *UNISA*

PARTICULARS OF ANIMALS WHICH MAY BE ~~HUNTED~~/CATCH / COLLECTED

Number	Species	Scientific name
	Namaqua rock mouse	*Micaelamys namaquensis*
	Red veld rat	*Aethomys chrysophilus*
	Pigmy mouse	*Mus minutoides*
	Four-striped mouse	*Rhabdomys pumilio*
	Single-striped mouse	*Lemniscolmys rosalia*
	Grey climbing mouse	*Dendromus melanitis*
	Chestnut climbing mouse	*Dendromus mysticalis*
	Vlei rat	*Mys irroratisoto*
	Spiny mouse	*Aconys spinossimus*
	Multimammate mouse	*Mastomys coucha*
	Bushveld gerbil	*Gerbilliscus leucogaster*
	Highveld gerbil	*Gerbilliscus brantsi*
	Woodland doremouse	*Graphiurus murinus*
	Fat mouse	*Steatomys pratensis*
	Rock elephant shrew	*Elephantulus myurus*
	Reddish grey musk show	*Crocidura cyanae*
	Swamp musk shrew	*Crocidura mariquensis*
	Asian house shrew	*Suncus infinitessimus*

PLACE: Telperion Nature Reserve-**MPUMALANGA PROVINCE**

In terms of and subject to the provisions of the Nature Conservation Act (Act No. 10 Of 1998) and the regulations framed thereunder , the above-mentioned person is hereby authorised, subject to the conditions and requirements appearing and this permit, to ~~hunt~~/catch/collected and animals referred to above during the period of validity of this permit on behalf of the institution or department referred to above

1. **Species that are captured, be released as soon as possible after processing at the site of capture.**
2. **Any dead specimens must be deposited at a recognised National Museum.**
3. **A list of all species that are trapped and positively identified (with the relevant point locality data (GPS points), be forwarded to Terrestrial Ecosystems, Lydenburg.**
4. **Permit holder must obtain written permission from the legal owner or occupier of the land to conduct the research.**
5. **Copies of scientific reports or publications be sent to the MTPA, Terrestrial Unit, at the above address.**

Period of validity of permit: From date of issue to: *31 December 2017*

....
for CHIEF EXECUTIVE OFFICER Signature of permit holder

AA
12/1/2017

CONSERVATION SERVICES
Private Bag X11338 Mbombela, SOUTH AFRICA, 1200 Tel: +27 (13) 759 5300/1 Fax: +27 (13) 759 5490 www.mpumalanga.com

MPUMALANGA
THE PLACE OF THE RISING SUN

SEE CONDITIONS AND REQUIREMENTS ON REVERSE SIDE

Open Rubric
