# Supplementary material for: Impact of Seriphium plumosum densification on Mesic Highveld Grassland biodiversity in South Africa
Source: R Soc Open Sci. 2020 Apr 15;7(4):192025. doi: 10.1098/rsos.192025 (PMC7211835; doi:10.1098/rsos.192025)
Supplement: BirdLife SA Ethics [file rsos192025supp2.pdf]

**Project number:**

2016/05/B

# BIRDLIFE SOUTH AFRICA ETHICS COMMITTEE

## APPROVAL CERTIFICATE

[Decision of the BirdLife South Africa Ethics Committee (BLSAEC) for the use of animals for research purposes.]

| | |
|---|---|
| APPROVAL PERIOD: | 1/12/2016 – 1/10/2018 |
| PROJECT TITLE: | The effect of *Seriphium plumosum* on the biodiversity of grasslands at Telperion, Mpumalanga, South Africa. |
| PROJECT LEADER: | Susannah Patrocinio |
| CATEGORY: | B - Studies on vertebrate species that are expected to produce little or no discomfort |
| SPECIES OF ANIMAL: | Various bird and small mammal species as listed in the addendums to the application. |
| NUMBER OF ANIMALS: | *In situ* population |
| APPROVED: | Yes |
| NOT APPROVED: | |
| CONDITIONAL APPROVAL | No |
| CONDITIONS TO APPROVAL: (The following conditions must be met before commencement of the study.) | None. |
| REPORTING REQUIREMENTS | Report I – Annual Progress report;<br>Report G – Adverse Event report: as soon as the adverse event occurs;<br>Report H – Application for Modifications and Extensions: as and when required. |



Open Rubric

**Please note:**

1. Should the experimental procedure(s) or number of animals or species of animal required change, document "H. Application for modifications and extensions" should be submitted to the Committee for review and approval before commencement of the research. Feedback must be given to the Committee annually and at the completion of the study.
2. In the event of an adverse event/unintended impact, the BLSAEC administrator, Linda van den Heever, should be contacted immediately at linda.vdheever@birdlife.org.za, or +27 (11) 789 1122. Should she be unavailable, the BLSAEC veterinary representative and chairperson, Dr Brett Gardner, should be contacted at +27 (82) 265 3393.

**Signature:** ____ Smit-Robinson _ **Date:** _____**18 November 2016**_____

Dr Hanneline Smit-Robinson
(Acting Chairperson of the BirdLife South Africa Ethics Committee)
