# Supplementary material for: Impact of Seriphium plumosum densification on Mesic Highveld Grassland biodiversity in South Africa
Source: R Soc Open Sci. 2020 Apr 15;7(4):192025. doi: 10.1098/rsos.192025 (PMC7211835; doi:10.1098/rsos.192025)
Supplement: CAES Ethics [file rsos192025supp3.pdf]

UNISA university of south africa

UNISA CAES ANIMAL RESEARCH ETHICS REVIEW COMMITTEE

Date: 07/07/2017

NHREC Registration # :	REC-170616-051
ERC Reference # :	2017/CAES/096
Name :	Ms SC Patrocinio
Student #:	39609979

Dear Ms Patrocinio

Decision: Ethics Approval from 06/07/2017 to 31/07/2018

Researcher(s): Ms SC Patrocinio
patros@unisa.ac.za; (011) 471-2016

Supervisor (s): Prof LR Brown
lrbrown@unisa.ac.za; (011) 471-2339

Dr AS Barrett
barreas@unisa.ac.za; (011) 471-3592

Dr H Smit-Robinson
conservation@birdlife.org.za

Working title of research:

The effect of *Seriphium plumosum* densification on grassland biodiversity in Telperion Nature Reserve, Mpumalanga, South Africa

Qualification: MSc Nature Conservation

Thank you for the application for research ethics clearance by the Unisa CAES Animal Research Ethics Review Committee for the above mentioned research. Ethics approval is granted for a one-year period. After one year the researcher is required to submit a progress report, upon which the ethics clearance may be renewed for another year.

Due date for progress report: 31 July 2018

*The **low risk application** was **reviewed** by the CAES Animal Research Ethics Review Committee on 06 July 2017 in compliance with the Unisa Policy on Research Ethics and the Standard Operating Procedure on Research Ethics Risk Assessment.*

The proposed research may now commence with the provisions that:

1. The researcher(s) will ensure that the research project adheres to the values and principles expressed in the UNISA Policy on Research Ethics.
2. Any adverse circumstance arising in the undertaking of the research project that is relevant to the ethicality of the study should be communicated in writing to the Committee.
3. The researcher(s) will conduct the study according to the methods and procedures set out in the approved application.
4. Any changes that can affect the study-related risks for the research participants, particularly in terms of assurances made with regards to the protection of participants' privacy and the confidentiality of the data, should be reported to the Committee in writing, accompanied by a progress report.
5. The researcher will ensure that the research project adheres to any applicable national legislation, professional codes of conduct, institutional guidelines and scientific standards relevant to the specific field of study. Adherence to the following South African legislation is important, if applicable: Protection of Personal Information Act, no 4 of 2013; Children's act no 38 of 2005 and the National Health Act, no 61 of 2003.
6. Only de-identified research data may be used for secondary research purposes in future on condition that the research objectives are similar to those of the original research. Secondary use of identifiable human research data require additional ethics clearance.
7. No field work activities may continue after the expiry date. Submission of a completed research ethics progress report will constitute an application for renewal of Ethics Research Committee approval.

Note:

*The reference number **2017/CAES/096** should be clearly indicated on all forms of communication with the intended research participants, as well as with the Committee.*

University of South Africa
Preller Street, Muckleneuk Ridge, City of Tshwane
PO Box 392 UNISA 0003 South Africa
Telephone: +27 12 429 3111 Facsimile: +27 12 429 4150
www.unisa.ac.za

Yours sincerely,

Please note conditions

Prof MA Antwi
Acting Chair of CAES Animal ERC
E-mail: antwima@unisa.ac.za
Tel: (011) 670-9391

Prof MJ Linington
Executive Dean : CAES
E-mail: lininmj@unisa.ac.za
Tel: (011) 471-3806

University of South Africa
Preller Street, Muckleneuk Ridge, City of Tshwane
PO Box 392 UNISA 0003 South Africa
Telephone: +27 12 429 3111 Facsimile: +27 12 429 4150
www.unisa.ac.za
